# Spinal Alignment and Pain: An Assessment of Amateur Road Cyclists—A Pilot Study

**DOI:** 10.3390/healthcare13020129

**Published:** 2025-01-11

**Authors:** Wojciech Kasperek, Aleksandra Kielar, Mirosław Pasierb, Monika Vaskova, Beata Ružbarska, Wojciech Czarny, Mariusz Drużbicki

**Affiliations:** 1 Physiotherapy Department, Institute of Health Sciences, College of Medical Sciences, University of Rzeszów, 35-215 Rzeszow, Poland; mpasierb@ur.edu.pl (M.P.); mdruzbicki@ur.edu.pl (M.D.); 2 Institute of Medical Sciences, College of Medical Sciences, University of Rzeszów, 35-025 Rzeszow, Poland; alkielar@ur.edu.pl; 3 Faculty of Sports, University of Presov, 080-01 Presov, Slovakia; monika.vaskova@unipo.sk (M.V.); beata.ruzbarska@unipo.sk (B.R.); 4 Institute of Physical Culture Sciences, College of Medical Sciences, University of Rzeszów, 35-010 Rzeszow, Poland; wczarny@ur.edu.pl

**Keywords:** low back pain, posture, biomechanics, physical activity, cycling

## Abstract

Background: Cycling involves specific body positions that, when maintained for prolonged periods, may affect spinal curvature and increase the risk of pain-related issues. This study aimed to evaluate sagittal spinal curvatures, the prevalence of pain in spinal segments, and their interrelation among amateur road cyclists. Methoods: The research included 30 male participants aged 18–48 years. Pain severity was assessed using the Visual Analog Scale (VAS) and Laitinen scale, while spinal curvature was evaluated with an electronic inclinometer. Results: Results showed no statistically significant differences in spinal curvature angles between cyclists with and without pain complaints (*p* = 0.056). However, tendencies were noted, such as higher mean VAS scores for lower back pain (4.90) compared to neck pain (3.38), and variations in parameters like Beta, LL, and KGP. Conclusions: While the findings did not confirm clear distinctions, they suggest trends indicating potential links between spinal curvatures and pain occurrence. These results underscore the importance of further studies involving larger cohorts to verify these observations and explore the biomechanical adaptations associated with amateur cycling. Insights from such research could inform strategies for preventing and managing spinal pain among cyclists.

## 1. Introduction

Cycling is a globally popular physical activity due to its health, environmental, and transportation benefits [1,2]. This discipline encompasses various types of cycling, including road, track, and mountain biking. Each type, particularly road cycling, is characterized by a specific body position, involving forward leaning and intense engagement of the gluteal muscles, which exert constant pressure on the structures of the lower spine [2]. Strain arising from maintaining this specific posture is often a consequence of equipment not being adjusted to the cyclist’s body dimensions and physical conditions [3]. Additionally, improper or absent warm-up routines serve as an additional risk factor [4]. Muscle activation asymmetries also constitute a risk factor for the occurrence of spinal pain. Researchers highlight the association between a reduction in the thickness and cross-sectional area of the transversus abdominis muscle and the lumbar portion of the multifidus muscle with lower muscle endurance and lumbar spine pain [5,6]. Consequently, both professional and amateur cyclists may experience spinal pain of varying intensity.

Cycling as a sport has the highest absolute number of annual injuries [7]. Road cycling positions are optimized to enhance speed and efficiency by achieving proper aerodynamics [8]. A stable and strong torso is essential for optimal power transfer, and the correct adjustment of bike components significantly impacts the cyclist’s biomechanics and efficiency [9]. Key bike elements directly affecting the cyclist’s spine include handlebars, the saddle, and crank arm length. In road cycling, three basic handlebar grip positions are distinguished: hoods position, drops position, and tops position. While riding in the hoods position, the cyclist’s torso is inclined at approximately 45 degrees, whereas in the drops position, the inclination increases to about 60 degrees [10]. The handlebar angle is individually adjusted for the cyclist. Excessive inclination forces the rider into a more pronounced forward lean, increasing pressure on the hands and reducing the effectiveness of the core muscles, ultimately decreasing power generation and transfer efficiency [11,12]. The bicycle saddle plays a pivotal role in rider comfort and cycling efficiency. Saddle width should be tailored to the cyclist’s ischial tuberosity width [13]. Optimal saddle height ensures 30 degrees of knee extension at the lowest pedal position while maintaining stable pelvic positioning on the saddle [14]. Additionally, the ankle joint angle should range between 0 and 18 degrees of plantar flexion [15]. The saddle can be tilted downward by 1–6 mm; a greater tilt can lead to discomfort and increased strain on the arms. Excessive rearward saddle displacement increases the load on the gluteal muscles and the hamstring muscle group, potentially causing pain in the pelvic region, spine, and lower limbs. Conversely, excessive forward saddle displacement overburdens the quadriceps and increases knee joint flexion angles [16]. Crank arm length affects joint mobility and spinal alignment during limb movement. Longer crank arms may cause increased external rotation of the knee joint, posterior pelvic tilt, and excessive lumbar spine curvature [17]. A literature review reveals that spinal pain in athletes and non-athletes is widely discussed [18]. However, most studies addressing this issue focus on amateurs practicing sports such as football, swimming, volleyball, basketball, tennis, dancing, or sailing [19,20,21,22]. To the best of the authors’ knowledge, there is a limited scientific literature evaluating the prevalence and prevention of spinal pain in both professional and amateur road cyclists. This pilot study aims to assess the pain symptoms and spinal curvature configuration in the sagittal plane, as well as the relationship between these factors depending on the location of pain among male amateur cyclists in Poland.

## 2. Materials and Methods

### 2.1. Study Participants

This pilot study involved 30 men aged 18–50 years, residing in southeastern Poland, who were amateur road cyclists. The inclusion criteria for the study were as follows: (1) written informed consent to participate in the study, (2) aged between 18 and 50 years, (3) engagement in cycling training at least twice per week, and (4) absence of comorbidities or surgeries within 12 months prior to the assessment. The exclusion criteria were: (1) professional practice of road cycling, (2) professional practice of other cycling, and (3) professional practice of other sports. Based on the analysis of a short demographic questionnaire of their own making, participants were divided into three groups: a group with no pain (control group, CG), which included 12 individuals, the group reporting cervical spine pain symptoms (CSP), which included 8 individuals, and the group reporting lumbar spine pain symptoms (LSP), which included 12 individuals. The characteristics of the study groups are presented in Table 1.

### 2.2. Study Procedures

All research procedures were conducted between May 2022 and October 2022, during the racing season. Participants were recruited from amateur cyclists competing in races organized in southeastern Poland. The study was performed before cycling training sessions, between 8:00 a.m. and 11:00 a.m. The research procedures included a short author questionnaire, physical examination of the anteroposterior spinal curvatures using an electronic inclinometer, and the Laitinen questionnaire. The author questionnaire included questions about basic epidemiological data, the presence and localization of spinal pain, and its intensity, assessed using the Visual Analog Scale (VAS). The VAS is a widely utilized tool for evaluating the severity of pain symptoms due to its simplicity, precision, and versatility. It is typically presented as a 10 cm line, with one end labeled as “no pain” and the other as “worst imaginable pain” [23]. The assessment of anterior–posterior spinal curvatures was conducted using an electronic inclinometer (Samsung M35G smartphone). The use of such mobile tools is becoming increasingly common among researchers due to their portability and capability to perform evaluations in training environments [24]. The application of this device, equipped with 3D accelerometers, digital magnetometric sensors, and gyroscopes, has been validated for reliability and test–retest validity, and it is recommended as an alternative tool to traditional assessment methods [24,25]. The spinal curvature measurements were performed in an upright standing position with fully extended lower limbs [24]. The examiner performed the following measurements: First measurement: The upper edge of the device was placed in the middle of the intervertebral space along the line connecting the posterior superior iliac spines—Alpha 1 angle (S1). Second measurement: The center of the phone was applied to the medial point of the sacral bone along the line connecting the so-called Venus dimples—Alpha 2 angle. Third measurement: The center of the device was placed at the thoracolumbar transition (Th/L)—Beta angle. Fourth measurement: The upper edge of the device was applied to the spinous process of C7—Gamma angle. Fifth measurement: The upper edge of the device was applied to the midpoint of thoracic kyphosis at the spinous process of Th3—Delta angle [26,27,28]. Spinal curvature changes were evaluated based on the methodology and guidelines for measurements described by Walicka et al. [27]. The normative range for the Alpha 1 angle is 15–30 degrees. The values of the Alpha 2 and Beta angles were summed to determine the lumbar lordosis angle (LL), with a normative range of 30–40 degrees. Additionally, the Beta and Gamma angles were summed to calculate the inclination of the upper thoracic kyphosis (UTK), with a normative range of 30–40 degrees [27]. The Laitinen scale was used for subjective assessment of pain levels. It consists of four indicators related to pain severity and frequency, the use of analgesics, and limitations in physical activity [29,30]. The study protocol was approved by the Bioethics Committee of the University of Rzeszów. All research procedures were conducted in accordance with the Declaration of Helsinki, and all participants provided written informed consent prior to the commencement of the study.

### 2.3. Statistical Analysis

The obtained results were subjected to statistical analysis using the Statistica software version 13.3. Quantitative variables were presented using descriptive statistics, including the mean, standard deviation, first quartile, median, third quartile, minimum, and maximum values. Nominal and ordinal variables were presented as absolute and relative frequencies. The normality of distribution within each group was assessed using the Shapiro–Wilk test. For data with a normal distribution, Student’s *t*-test was applied, whereas for data not meeting this assumption, the non-parametric Mann–Whitney U test was used. Correlations between parameters were calculated using Pearson’s correlation coefficient (r). A significance level of *p* < 0.05 was adopted.

## 3. Results

Approximately 60% of the surveyed cyclists reported experiencing pain symptoms. While the comparison of pain levels between the cervical and lumbar spine groups yielded a *p*-value of 0.056 slightly above the significance threshold of *p* < 0.05—indicating no statistically significant difference in pain levels between the groups—the test statistic (Z = 18) suggests a trend toward differences. However, this trend may not reach statistical significance due to the small sample size. Despite this, the descriptive statistics (means and medians) suggest that individuals with lumbar spine pain experience more intense symptoms compared to those with cervical spine pain, as detailed in Table 2.

The interpretation of data from the Laitinen scale indicated a milder nature of pain in the cervical spine compared to the lumbar spine. In both groups, intermittent pain predominated; however, in 20% of cases, the lumbar spine exhibited very frequent pain. Most participants in both groups did not use analgesics. Nevertheless, the presence of lumbar spine pain was more frequently associated with limitations in physical activity compared to cervical spine pain. Detailed data are presented in Table 3.

The analysis of spinal segment inclinations in relation to pain occurrence did not reveal statistically significant differences, suggesting that the spinal segment inclinations in the group with lumbar pain do not significantly differ from those in the control group. Higher mean values for variables such as LL and upper thoracic kyphosis (KPG) in the study group may indicate a tendency toward greater inclination in this group, but these differences are too small to be considered significant. Detailed data are provided in Table 4.

The assessment of differences in spinal segment inclination between the control group and the group with cervical spine pain revealed that no variable fell below the 0.05 threshold, indicating that differences in spinal segment inclinations between the control group and the group with cervical spine pain are statistically insignificant. Detailed data values are presented in Table 5.

## 4. Discussion

In this pilot study, we evaluated the impact of amateur road cycling on spinal curvature and the frequency of pain in various spinal segments, as well as the correlations between these factors. We aimed to compare these parameters across distinct groups. Our assessment revealed that pain was reported by 60% of the studied cyclists, with varying localizations. Similar findings were reported by Battista et al. among amateur cyclists in Italy, where spinal pain was noted in 26.5% of participants within the last 12 months [31]. Piotrowska et al. also confirmed the occurrence of spinal pain in 42% of amateur cyclists participating in cross-country and marathon races on mountain or cyclocross bikes in Poland [32]. Teyeme et al. found that the most common pain symptom among both professional and amateur cyclists was lower back pain (LBP), reported by 26% of respondents, while cervical spine discomfort was noted by 9% [2]. According to Schultz et al., recreational cyclists riding 160 km or more per week also reported a significantly higher prevalence of LBP [33]. These findings align with our observations, as in our study, cyclists more frequently reported pain in the lumbar spine. The mean pain intensity among our participants was higher in the group with lumbar pain (4.90) compared to the group with cervical spine pain (3.38). There is a lack of literature comparing spinal curvatures in amateur cyclists with and without spinal pain. Although this topic remains unexplored, spinal curvature adaptations related to different sports disciplines have been described by several authors. In our study, the assessment of spinal curvature revealed no statistically significant differences, suggesting that the inclinations of spinal segments in the groups with lumbar or cervical pain did not differ significantly from the control group. However, the observed higher means in the cervical pain group (e.g., Gamma and Beta) and the lumbar pain group (e.g., LL and KGP) may indicate a tendency toward increased spinal segment inclinations. Muyor et al. compared spinal curvature among road cyclists, mountain bikers, and non-cyclists, reporting that road cyclists had significantly greater thoracic kyphosis compared to the other groups [34]. Boroojerdi et al. examined elite and speed-class Iranian cyclists and found that both groups exhibited significantly increased thoracic kyphosis compared to non-cycling controls, while lumbar lordosis remained within normal ranges in all groups [35]. Similarly, in Muyor et al.’s study comparing professional cyclists with non-athletes, the inclination of thoracic kyphosis was significantly greater in cyclists than in controls [36]. Alricsson et al. demonstrated that young professional cross-country skiers showed a significant increase in thoracic kyphosis and lumbar lordosis after five years of training, with a higher prevalence of LBP among those with more pronounced curvatures [37]. The studies mentioned above indicate that road cyclists are characterized by altered spinal curvatures, which may contribute to an increased incidence of spinal pain. From a practical and clinical perspective, understanding the relationship between changes in spinal curvature and pain can help optimize training methods and recovery cycles, raising awareness among coaching staff about these pathologies. The main limitation of our study is the small sample size, the lack of female athletes and the small region in which the study participants were qualified, so the results should be interpreted with caution. Future studies must take into account these limitations and be expanded to include more age groups.

## 5. Conclusions

Back pain is a prevalent issue among amateur cyclists, with moderate to severe lower back pain being particularly common. The lack of significant differences in spinal curvature between cyclists with and without pain suggests that spinal alignment alone may not be the primary contributing factor. These findings highlight the potential importance of other influences, such as biomechanical, ergonomic, or training-related factors, in the development of back pain. To draw more robust conclusions, further studies involving larger populations and a comprehensive analysis of both intrinsic and extrinsic risk factors are needed.

## Figures and Tables

**Table 1:** Study groups’ characteristics.

Variable	Group	x	SD	Q1	Me	Q3	Min	Max
Age	CG	31.8	9.42	23.00	33.00	40.00	18.00	44.00
	LSP	30.90	8.05	25.00	29.50	36.00	21.00	48.00
	CSP	30.25	6.65	25.00	28.50	35.50	23.00	41.00
	Total	31.10	8.05	25.00	31.00	38.00	18.00	48.00
Height [CM]	CG	180.25	6.21	176.00	180.00	186.00	171.00	190.00
	LSP	179.70	3.86	178.00	180.00	183.00	172.00	186.00
	CSP	171.63	6.20	170.00	172.50	176.50	158.00	177.00
	Total	177.77	6.54	173.00	178.00	182.00	178.00	190.00
Body mass [KG]	CG	72.83	10.40	71.00	73.50	78.00	51.00	87.00
	LSP	81.00	9.83	72.00	82.50	90.00	68.00	94.00
	CSP	67.63	7.93	60.00	70.00	72.00	57.00	80.00
	Total	74.17	10.71	70.00	73.50	82.00	51.00	94.00
BMI	CG	22.40	3.03	20.69	21.95	23.80	17.44	28.10
	LSP	25.07	2.77	22.72	25.90	27.17	20.31	28.70
	CSP	22.92	1.97	21.56	23.13	24.38	19.72	25.50
	Total	23.43	2.87	21.10	22.85	25.62	17.44	28.70

**Table 2:** Comparison of pain complaints on the VAS.

Variable	Group	n	x ± SD	Me	Min	Max	Z	*p*
Pain	CSP	8	3.38 ± 2.26	2.00	2.00	7.00	18.00	0.056
	LSP	10	4.90 ± 2.42	4.00	3.00	10.00		

**Table 3:** Laitinen scale pain scores in the cervical and lumbar spine in the study group of cyclists.

Laitinen Pain Scales					
Variable		CSP		LSP	
		n	%	n	%
Pain intensity	Mild	6	25.00	6	60.00
	Strong	2	75.00	3	30.00
	Very strong	0	0	1	10.00
	Not sustainable	0	0	0	0
Pain frequency	Periodically	7	87.50	8	80.00
	Frequent	1	12.50	0	0
	Very frequent	0	0	2	20.00
	Continuous	0	0	0	0
Painkillers intake	Without medication	6	75.00	8	80.00
	Periodically	2	25.00	2	20.00
	Permanently small doses	0	0	0	0
	Permanently big doses	0	0	0	0
	Permanently very big doses	0	0	0	0
Motor activity limitation	None	7	87.50	5	50.00
	Partial	1	12.50	4	40.00
	Making work difficult Making work impossible	0	0	1	10.00
		0	0	0	0

**Table 4:** Differences in spinal segment inclination between the control group and the group with lumbar spine pain.

	CG			LSP			
	x	Me	SD	x	Me	SD	*p*
Alfa 1	22.67	23.50	5.43	24.40	24.00	3.66	0.4010
Alfa 2	16.75	16.00	5.66	16.70	17.00	4.79	0.9826
Beta	8.42	10.00	4.60	11.20	10.00	5.51	0.2112
Gamma	29.75	30.00	4.52	29.40	29.00	4.62	0.8597
Delta	19.17	21.00	4.84	19.80	19.50	5.01	0.7666
LL	25.17	25.50	3.74	27.90	27.50	5.80	0.1965
KPG	38.17	38.50	7.92	40.60	42.00	8.00	0.4833

**Table 5:** Differences in spinal segment inclination between the control group and the group with cervical spine pain.

	CG			LSP			
	x	Me	SD	x	Me	SD	*p*
Alfa 1	22.67	23.50	5.43	25.00	24.50	3.12	0.2882 ^1^
Alfa 2	16.75	16.00	5.66	17.13	15.50	4.67	0.8785 ^1^
Beta	8.42	10.00	4.60	10.75	12.00	4.71	0.2856 ^1^
Gamma	29.75	30.00	4.52	32.13	34.00	6.69	0.3534 ^1^
Delta	19.17	21.00	4.84	19.38	20.50	4.81	0.9257 ^1^
LL	25.17	25.50	3.74	27.88	28.00	4.94	0.1793 ^1^
KPG	38.17	38.50	7.92	42.88	45.00	9.11	0.2318 ^2^

^1^ t-Student; ^2^ U-Mann–Whitney.

## Data Availability

The datasets used and/or analyzed during the current study are available from the corresponding author on reasonable request.
